# The Effects of Acute Virtual Reality Exergaming on Mood and Executive Function: Exploratory Crossover Trial

**DOI:** 10.2196/38200

**Published:** 2022-09-28

**Authors:** Genta Ochi, Ryuta Kuwamizu, Tomomi Fujimoto, Koyuki Ikarashi, Koya Yamashiro, Daisuke Sato

**Affiliations:** 1 Department of Health and Sports Niigata University of Health and Welfare Niigata Japan; 2 Institute for Human Movement and Medical Sciences Niigata University of Health and Welfare Niigata Japan; 3 Laboratory of Exercise Biochemistry and Neuroendocrinology Faculty of Health and Sport Sciences University of Tsukuba Tsukuba Japan; 4 Major of Health and Welfare Graduate School of Niigata University of Health and Welfare Niigata Japan

**Keywords:** virtual reality, exergaming, exercise, executive function, physical activity, mental health

## Abstract

**Background:**

Virtual reality (VR) exergaming is a new intervention strategy to help humans engage in physical activity to enhance mood. VR exergaming may improve both mood and executive function by acting on the prefrontal cortex, expanding the potential benefits. However, the impact of VR exergaming on executive function has not been fully investigated, and associated intervention strategies have not yet been established.

**Objective:**

This study aims to investigate the effects of 10 minutes of VR exergaming on mood and executive function.

**Methods:**

A total of 12 participants played the exergame “FitXR” under 3 conditions: (1) a VR exergame condition (ie, exercise with a head-mounted display condition [VR-EX]) in which they played using a head-mounted display, (2) playing the exergame in front of a flat display (2D-EX), and (3) a resting condition in which they sat in a chair. The color-word Stroop task (CWST), which assesses executive function; the short form of the Profile of Mood States second edition (POMS2); and the short form of the Two-Dimensional Mood Scale (TDMS), which assess mood, were administered before and after the exercise or rest conditions.

**Results:**

The VR-EX condition increased the POMS2 vigor activity score (rest and VR-EX: t_11_=3.69, *P*=.003) as well as the TDMS arousal (rest vs 2D-EX: t_11_=5.34, *P*<.001; rest vs VR-EX: t_11_=5.99, *P*<.001; 2D-EX vs VR-EX: t_11_=3.02, *P*=.01) and vitality scores (rest vs 2D-EX: t_11_=3.74, *P*=.007; rest vs VR-EX: t_11_=4.84, *P*=.002; 2D-EX vs VR-EX: t_11_=3.53, *P*=.006), suggesting that VR exergaming enhanced mood. Conversely, there was no effect on CWST performance in either the 2D-EX or VR-EX conditions. Interestingly, the VR-EX condition showed a significant positive correlation between changes in CWST arousal and reaction time (*r*=0.58, *P*=.046). This suggests that the effect of exergaming on improving executive function may disappear under an excessively increased arousal level in VR exergaming.

**Conclusions:**

Our findings showed that 10 minutes of VR exergaming enhanced mood but did not affect executive function. This suggests that some VR content may increase cognitive demands, leading to psychological fatigue and cognitive decline as an individual approaches the limits of available attentional capacity. Future research must examine the combination of exercise and VR that enhances both brain function and mood.

## Introduction

Many previous studies support the benefits of physical activity for improving physical and mental health. Physical activity has been shown to decrease the risk of noncommunicable diseases, such as cardiovascular disease and type 2 diabetes [1], and to improve mental health [2,3]. Furthermore, in both older and younger adults, high aerobic fitness has been reported to be beneficial for maintaining executive functions (mental set shifting, information updating, and inhibition of prepotent responses [4]), making physical activity increasingly important [5-8]. However, according to the World Health Organization, approximately 25% of adults and 80% of adolescents worldwide are inactive, making physical inactivity a serious health problem. In addition, the global COVID-19 outbreak has forced people to socially disperse and self-isolate to prevent the spread of the infection, resulting in a greater proportion of individuals experiencing physical inactivity [9].

Recently, exergaming using virtual reality (VR) has been the focus of a new approach to promote physical activity [10]. The main feature of VR is that it combines a realistic 3D environment, body tracking using a head-mounted display (HMD), and handheld controllers to immerse the user in a virtual simulation [11,12]. Previous studies have suggested that VR may increase the potential for long-term participation in physical activity by distracting attention from negative images of exercise that depict it as physically fatiguing [13], boring, and strenuous [14,15], and inducing a positive mood toward exercise [16-18]. Dopaminergic nervous system involvement has been postulated as a potential brain mechanism for this positive mood effect, and VR has been used to rehabilitate patients with Parkinson disease, which is associated with decreased dopamine levels [19,20]. Previous studies have also shown that the brain’s dopaminergic system, originating in the ventral tegmental area and the substantia nigra, is related to executive function through the prefrontal cortex and the striatum [21-23]. This suggests that VR exergaming may enhance executive functions governed by the prefrontal cortex, which may further augment VR’s value as an exercise prescription. However, the effects on the executive function of acute exercise under VR have not been fully tested, and optimal levels of VR use remain unknown.

To examine the effects of acute exercise under VR on mood and executive function, an experimental model with high reproducibility is required to assess mood and executive function. In this study, we used the color-word Stroop task (CWST) to assess executive function, and the Two-Dimensional Mood Scale (TDMS) to assess mood state (arousal, pleasure, vitality, and stability). Previous studies have used these measures to examine exercise effects, and have found that 10 minutes of low- or moderate-intensity exercise enhanced mood states, such as arousal and pleasure, and executive function [24,25]. Furthermore, in our prior research, we identified the effects of different exercise conditions, such as listening to music [26] and a hypoxic environment [27], on mood and executive function. The 10-minute exercise set used in these studies provides an excellent model for examining VR exercises’ effects on mood and executive function.

Previous studies suggested that 10 minutes of exercise enhances executive function [24,25,28-30], and that positive mood during exercise may influence the positive effect on executive function [25,26]. In this study, we hypothesized that VR is an environmental factor that enhances positive mood and executive function by exercise and aimed to clarify whether transient exercise under VR enhances both mood and executive function.

## Methods

### Experimental Procedure

Prior to beginning the main experimental process, each participant was given verbal instructions, guided through the consent process, practiced the CWST 3 times, and engaged in VR exergaming. Participants were instructed to play the in-game tutorial to learn how the game is played. Once the tutorial was completed, participants played the 10-minute program used in this experiment once to familiarize themselves with exergaming.

**Figure 1:**
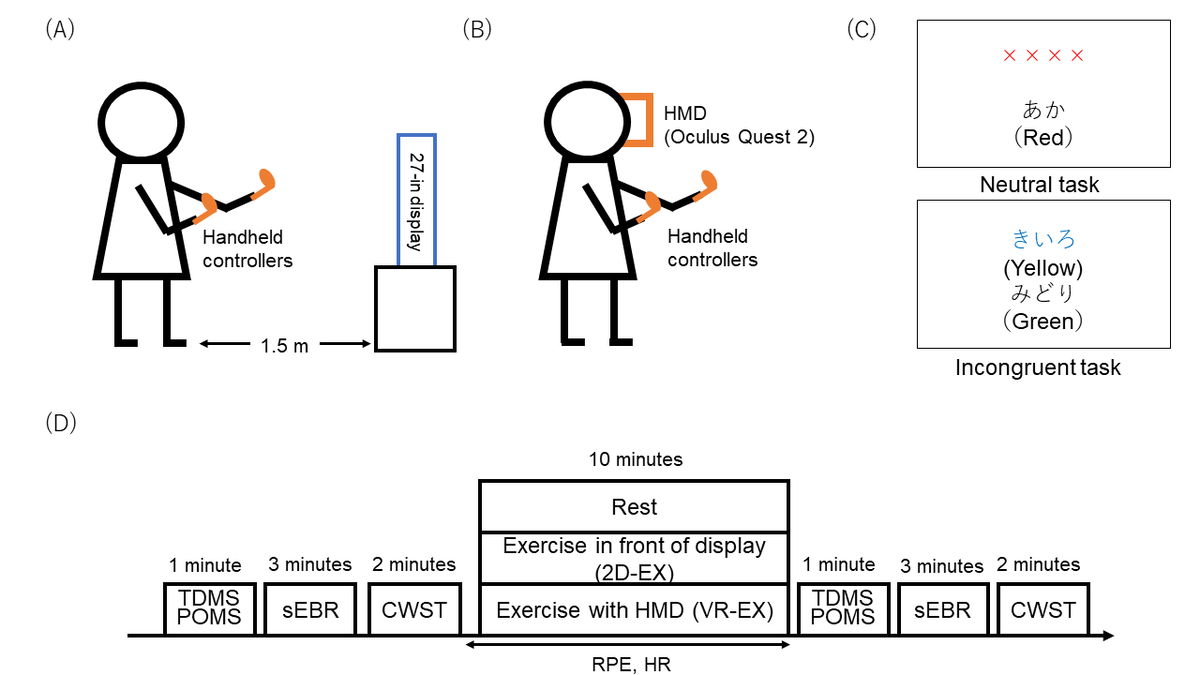
(A) The 2D-EX condition and (B) the VR-EX (HMD) condition. Participants performed exergaming, such as punching a target or avoiding obstacles on the display or HMD. (C) The CWST. Examples of single trials for the neutral and incongruent CWST trials presented in Japanese (English translations are in parentheses). (D) Psychological questionnaires, sEBR, and CWST were measured before and after 10 minutes of exercise or rest. 2D-EX: exercise in front of a display condition; CWST: color-word Stroop task; HMD: head-mounted display; POMS: Profile of Mood States; sEBR: spontaneous eye blink rate; TDMS: Two-Dimensional Mood Scale; VR-EX: exercise with a head-mounted display condition.

A few days after the first visit, participants engaged in 1 of the 3 experimental conditions—rest, exercise in front of a display condition (2D-EX), or exercise with an HMD (VR-EX). All participants completed all 3 conditions, each on a separate day, with the order counterbalanced across participants (Figure 1D). In the 2D-EX and VR-EX conditions, participants completed the CWST before and after 10 minutes of exercise. In the rest condition, participants completed the CWST before and after sitting in a chair for 10 minutes. In all conditions, participants completed a questionnaire and had their blink rate measured for 3 minutes before performing the CWST.

### Participants

A total of 13 right-handed Japanese-speaking young adults (7 men and 6 women) were recruited from June to July 2021. The sample size was determined by assuming that the effects of exercise would be similar to those found in our previous studies [27,31]. All participants were without regular exercise habits, Japanese native speakers, healthy, and unaware of the experimental procedures for which they volunteered. No participant reported a history of neurological, psychiatric, or respiratory disorders, and none had a condition requiring medical care. One male participant withdrew from the study because he was color-blind and had difficulty discriminating colors in executive function tasks. The remaining 12 participants (6 men and 6 women) were included in the main analyses (mean age 20.15 years, SD 3.05 years); 8 participants (67%) self-reported that they played computer games on a weekly or monthly basis, and 4 participants did not specify this information. All participants were asked to refrain from exercise, alcohol consumption, and caffeine for at least 24 hours prior to each experimental session to control for outside factors that could affect cardiovascular and executive functions. Post hoc sensitivity analysis performed based on this sample with 80% power and α=.05 demonstrated sufficient sensitivity to detect repeated-measures effects exceeding *f*=0.50 and paired *t* test differences exceeding *d*=0.85 (with a 2-tailed α), as computed using G*Power (3.1.9.2; The G*Power Team).

### Materials and Apparatus

#### Virtual Reality Setup

The FitXR exergame (developed by FITAR LIMITED) was administered using a commercially available HMD (Oculus Quest 2; Meta Platforms, Inc.). FitXR was selected because it is easy to play even for users with no previous exposure to VR, and can be continued for 10 minutes without interruption even if a play error occurs during the game. Using motion tracking, FitXR simulates handheld controllers as boxing gloves, whereby users punch the targets and must actively crouch and dodge obstacles. The game immerses the participant through tactile sensations of punching the target, auditory sensations of music playing, and performance feedback. In the VR-EX condition, participants played FitXR using an HMD and handheld controllers (Figure 1A).

#### Movie Setup for Exercise Conditions

For the same exercise condition without VR exposure (ie, the 2D-EX condition), participants exercised while watching the exergaming program movie displayed on a flat screen (27-in., 1920 × 1080 pixels, 60 Hz) positioned 1.5 m away from them (Figure 1B). This allowed participants to perform the same exercise as in the VR condition, but without the HMD. Although they performed the exercise with hand controllers in this condition, they did not receive tactile, auditory, or performance feedback.

#### Behavioral Measurements

The CWST, created from web-building platforms (Lab.js v19.1.0) [32], was used to evaluate executive function, and was adopted in an event-related design [8,24-30,33-35]. Two rows containing letters or words were presented on a screen and participants were instructed to decide whether the color in which the letters or words in the top row were written corresponded to the color name presented in the bottom row (Figure 1C), pressing a “yes” or “no” button with their right or left forefinger, respectively, to respond. Reaction time (RT) and correct rate were recorded for task performance.

The CWST comprised 3 trials: 16 neutral, 16 congruent, and 16 incongruent. Each task was presented using the same method as in our previous study [27,35], and participants were asked to judge whether the color of the color name word or symbol displayed in the upper row matched the meaning of the color name word displayed in the lower row. The correct answer ratio assigned to “yes” and “no” was 50%. Each stimulus was separated by an interstimulus interval showing a fixation cross for 2 seconds to avoid a prediction of the subsequent trial’s timing. The stimulus remained on the screen until a response was given or for 1 second. This study adopted Stroop interference, a specifically defined cognitive process, to elucidate the effect of an acute bout of exercise on executive function. We measured this by calculating the incongruent-neutral contrast, which is assumed to represent Stroop interference.

#### Physiological Measurements

Heart rate (HR) was measured with an HR sensor (H10; Polar Electro) and an HR monitor (Vantage V2; Polar Electro). Spontaneous eye blink rate (sEBR) was measured as a noninvasive brain dopaminergic system indicator [36-39]. Participants sat in front of a 27-inch display, located 70 cm from them, and were asked to look at a fixation cross presented at the center of the display at rest; the sEBR (per minute) was recorded for 3 minutes using a camcorder (120 frames/second, 2560 × 1440 pixels; Hero 8; GoPro, Inc.) that was set below the display. The sEBR was counted by 1 (GO) rater. Preliminarily, half the sample was counted by an independent rater (RK) and high validity was confirmed (r=0.995). The individual sEBR was calculated by dividing the total number of eye blinks during the 3-minute measurement interval by 3. All sEBR data were collected by 6 PM because sEBR can be less stable at night [37].

#### Psychological Measurements

Participant Rating of Perceived Exertion (RPE) [40] was recorded before and after the exercise intervention to assess psychological exercise intensity. In addition, the Japanese versions of the Profile of Mood States second edition (POMS2) [41] and TDMS questionnaires were administered to assess psychological indicators before and after the exercise intervention.

The POMS2 contains 35 items and evaluates 7 mood states (anger-hostility, confusion-bewilderment, depression-ejection, fatigue-inertia, tension-anxiety, vigor-activity, and friendliness). This study used a subset of 10 items related to vigor-activity and fatigue-inertia to verify whether exergaming enhances mood or causes fatigue.

The TDMS [42] is a momentary mood scale; the short form comprises 2 words describing arousal and pleasure states (lively and relaxed) and evaluates 4 mood states (arousal, pleasure, vitality, and stability). Participants were asked to indicate how they were feeling for each mood-expressing word using an 11-point Linkert scale that ranged from –5 (Listless) to 5 (Lively) and –5 (Irritated) to 5 (Relaxed). In addition to “words” and “numbers” describing the psychological state, the shortened version used “person illustrations” and “color images” to reduce the burden of answering for participants who were unfamiliar with the experiment.

### Statistical Analysis

All analyses were performed using R software (4.1.2; R Foundation for Statistical Computing) and the EZR on R Commander package [43]. Two-way repeated measures ANOVA tests were performed to compare between conditions. First, the Mendoza multisample sphericity test was used to assess whether sphericity was maintained. When the sphericity assumption was confirmed, we conducted a repeated measures ANOVA with Greenhouse-Geisser epsilon correction; otherwise we performed a repeated measures ANOVA. Significant differences obtained from the ANOVA were tested using the corresponding *t* test (paired) with Holm correction. To clarify the relationships between parameters and executive performance, we conducted Pearson correlation analyses. Statistical significance was set a priori at *P*<.05 for all comparisons.

### Ethics Approval

This study was conducted in accordance with the Declaration of Helsinki and was approved by the Ethics Committee of the Niigata University of Health and Welfare, Niigata, Japan (approval number 18631-210601). Participants were informed that their data would be kept confidential and provided written informed consent to participate in the study.

## Results

### Overview

All participants performed the experiment perfectly; no participant reported VR-related adverse effects such as motion sickness, dizziness, and headaches after VR-EX.

### Physiological Parameters

Table 1 summarizes the HR, RPE, and sEBR results. These were included in the repeated measures two-way ANOVA with condition (rest/2D-EX/VR-EX) and time (before/after) as within-participant factors. The results showed a significant interaction between condition and time in HR and RPE (HR: *F*_2,22_=41.9, *P*<.001; RPE: *F*_2,22_=39.5, *P*<.001) and a significant main effect of time (*F*_1,11_=16.1, *P*=.002) in sEBR. The paired *t* test with Holm correction showed significant differences in HR between the rest and 2D-EX conditions, and between the rest and VR-EX conditions during exercise (rest vs 2D-EX: t_11_=9.60, *P*<.001; rest vs VR-EX: t_11_=7.70, *P*<.001). The paired *t* test with Holm correction results showed significant RPE differences among all postexercise conditions (rest vs 2D-EX: t_11_=6.25, *P*<.001; rest vs VR-EX: t_11_=7.41, *P*<.001; 2D-EX vs VR-EX: t_11_=2.99, *P*=.01).

**Table 1:** Physiological parameters.

Conditions and variables		Before exercise, mean (SD)	During exercise, mean (SD)	After exercise, mean (SD)
**Rest**				
	HR^a^ (bpm)	74.7 (13.7)	70.3 (13.1)	N/A^b^
	RPE^c^ (point)	6.0 (0.0)	N/A	6.1 (0.3)
	sEBR^d^ (per minute)	29.9 (20.1)	N/A	36.0 (18.5)
**2D-EX^e^**				
	HR (bpm)	82.4 (8.8)	108.2 (15.0)^f^	N/A
	RPE (point)	6.0 (0.0)	N/A	10.1 (2.3)^f^
	sEBR (per minute)	30.7 (18.7)	N/A	41.2 (21.6)
**VR-EX^g^**				
	HR (bpm)	81.0 (10.0)	117.5 (18.7)^f^	N/A
	RPE (point)	6.0 (0.0)	N/A	11.7 (2.8)^f,h^
	sEBR (per minute)	34.0 (21.5)	N/A	38.1 (29.2)

^a^HR: heart rate.

^b^N/A: not applicable.

^c^RPE: Rating of Perceived Exertion.

^d^sEBR: spontaneous eye blink rate.

^e^2D-EX: exercise in front of a display condition.

^f^*P<.*05 versus rest.

^g^VR-EX: exercise with a head-mounted display condition.

^h^*P<.*05 versus 2D-EX.

### Psychological Parameters

**Figure 2:**
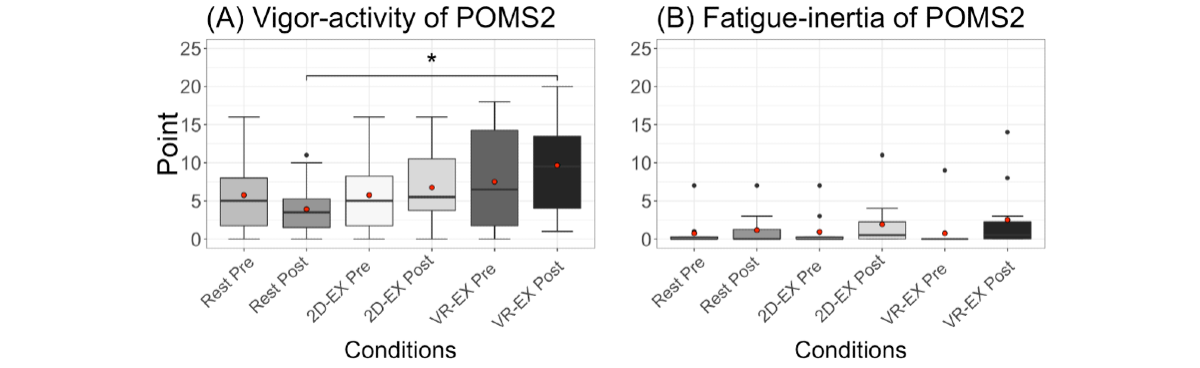
(A) Vigor-activity and (B) fatigue-inertia of POMS2 under each condition. The tops and bottoms of the boxes are third and first quartiles, respectively. The upper and lower ends of the whiskers represent the highest data points within 1.5 IQRs of the upper quartiles and the lowest data points within 1.5 IQRs of the lower quartiles, respectively. The bands inside the boxes indicate medians. The red circle is the mean. *P<.05. 2D-EX: exercise in front of a display condition; POMS2: Profile of Mood States second edition; VR-EX: exercise with a head-mounted display condition.

The POMS2 vigor-activity and fatigue-inertia results are shown in Figure 2. The 2-way repeated measures ANOVA with condition (rest/2D-EX/VR-EX) and time (before/after) as within-participant factors showed a significant interaction between condition and time in vigor-activity (*F*_2,22_=4.2, *P*=.03). The paired *t* test with Holm correction results showed significant differences between the rest and VR-EX conditions at postsession (t_11_=3.69, *P*=.003).

**Figure 3:**
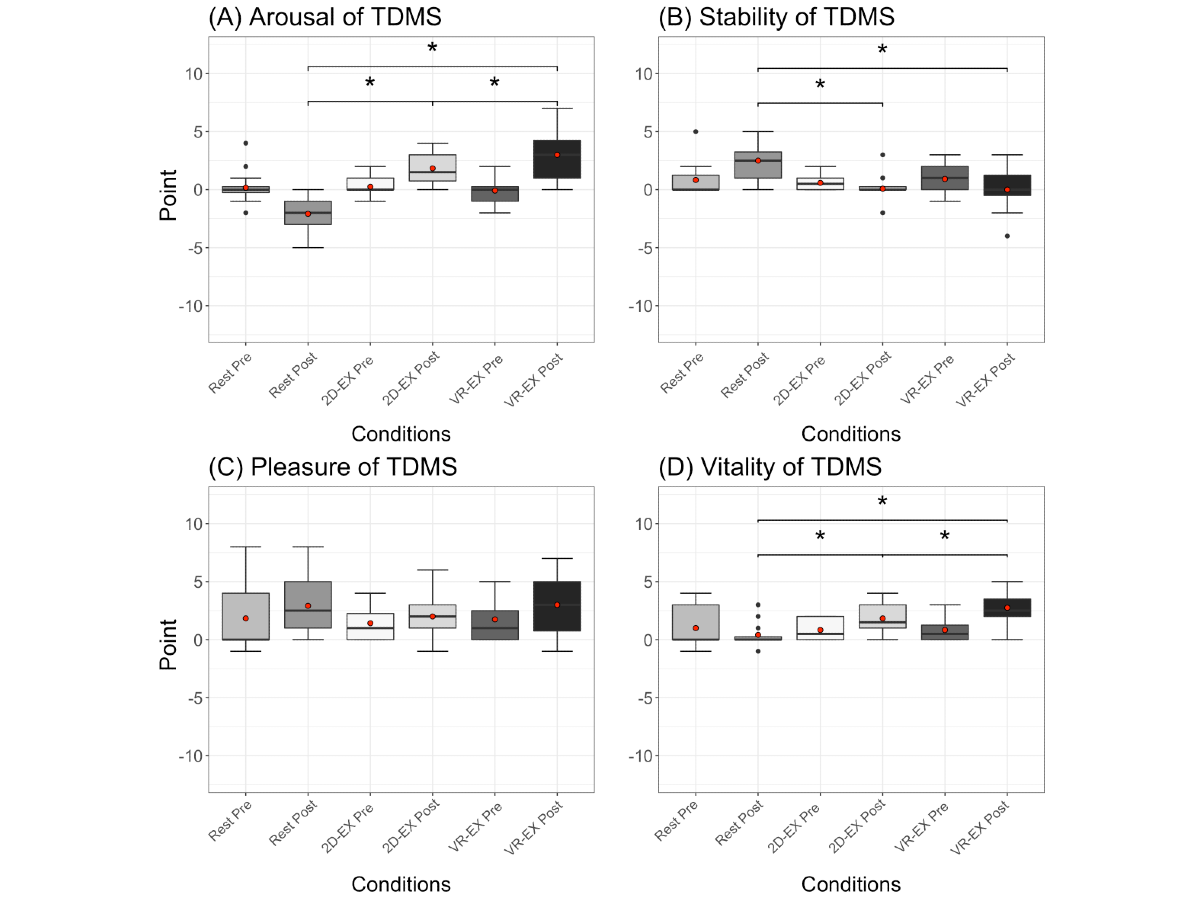
(A) Arousal, (B) pleasure, (C) vitality, and (D) stability of TDMS under each condition. The tops and bottoms of the boxes are third and first quartiles, respectively. The upper and lower ends of the whiskers represent the highest data points within 1.5 IQRs of the upper quartiles and the lowest data points within 1.5 IQRs of the lower quartiles, respectively. The bands inside the boxes indicate medians. The red circle is the mean. *P<.05. 2D-EX: exercise in front of a display condition; TDMS: Two-Dimensional Mood Scale; VR-EX: exercise with a head-mounted display condition.

The TDMS data in Figure 3 were analyzed using 2-way repeated measures ANOVA with condition (rest/2D-EX/VR-EX) and time (before/after) as within-participant factors, and showed a significant interaction between condition and time in arousal (*F*_1.26,13.87_=21.14, *P*<.001), stability (*F*_1.31,14.36_=12.34, *P*=.002), and vitality (*F*_1.42,15.67_=18.96, *P*<.001), and a significant main effect of time on pleasure (*F*_1,11_=8.99, *P*=.01). The paired *t* test with Holm correction results for arousal and vitality showed significant differences between the rest and 2D-EX conditions, between the rest and VR-EX conditions after exercise, and between the 2D-EX and VR-EX conditions after exercise (arousal—rest vs 2D-EX: t_11_=5.34, *P*<.001; rest vs VR-EX: t_11_=5.99, *P*<.001; 2D-EX vs VR-EX: t_11_=3.02, *P*=.01; vitality—rest vs 2D-EX: t_11_=3.74, *P*=.007; rest vs VR-EX: t_11_=4.84, *P*=.002; 2D-EX vs VR-EX: t_11_=3.53, *P*=.006). The paired t test with Holm correction results for stability showed significant differences between the rest and 2D-EX conditions (t_11_=3.82, *P*=.006), and between the rest and VR-EX conditions (t_11_=4.02, *P*=.006) after exercise.

### Executive Performance: Stroop Interference

Table 2 summarizes the RT and CWST results.

First, to examine whether a general CWST tendency could be reproduced in all conditions, RT and correct rate were included in a repeated measures ANOVA using the Greenhouse-Geisser correction, with trial (neutral/incongruent), condition (rest/2D-EX/VR-EX), and time (before/after) as within-participant factors. Results showed a significant main effect of trial on RT (*F*_1.24,13.65_=28.96, *P<*.001). There was no significant main effect or interaction in the correct rate (*F*_1.27,13.94_=3.50, *P*=.08). This might be because the percentage of correct answers remained high, above 90% (45.83/48, 95%), for all participants. Conversely, the RT results verified that Stroop interference was generally observed in all the sessions of this experiment. Thus, to clarify the effect of an acute bout of exercise on a specifically defined cognitive process, we analyzed Stroop interference (incongruent-neutral) by RT.

**Figure 4:**
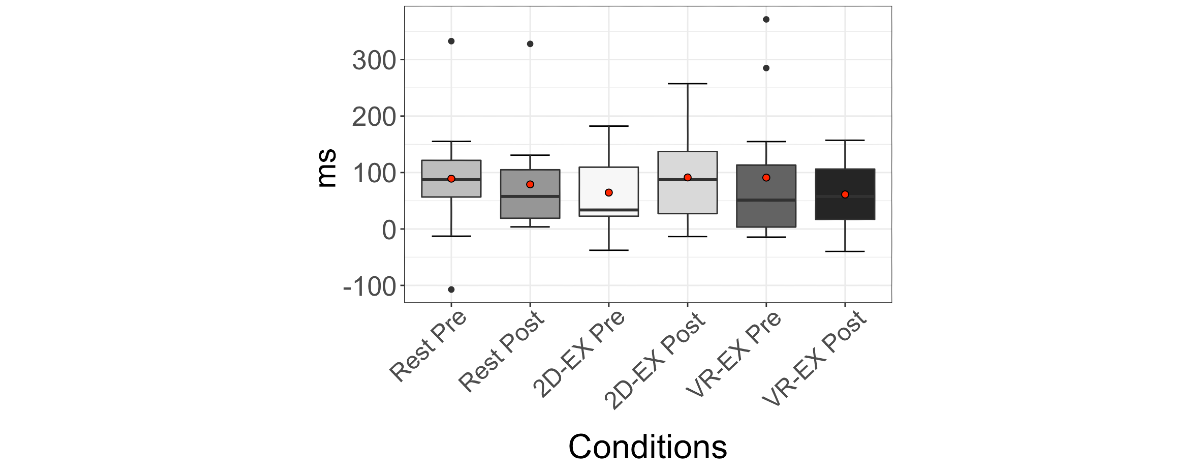
Stroop interference (RT) differences between pre- and postsessions for each condition. Stroop interference differences showed no significant main effect or interaction. The tops and bottoms of the boxes are third and first quartiles, respectively. The upper and lower ends of the whiskers represent the highest data points within 1.5 IQRs of the upper quartiles and the lowest data points within 1.5 IQRs of the lower quartiles, respectively. The bands inside the boxes indicate the medians. The red circle is the mean. 2D-EX: exercise in front of a display condition; RT: reaction time; VR-EX: exercise with a head-mounted display condition.

Next, to examine the RT interaction, we calculated the difference in the degree of Stroop interference between post- and presessions (incongruent-neutral presession, incongruent-neutral postsession). Then, the difference in the degree of Stroop interference between rest, 2D-EX, and VR-EX conditions was calculated using the Greenhouse-Geisser correction with condition (rest/2D-EX/VR-EX) and time (before/after) as within-participant factors. The results showed no significant main effect (condition: *P*=.95; time: *P*=.78) or interaction (*P*=.46; Figure 4).

**Table 2:** RT^a^ results and CWST^b^ correct rate.

Variable and conditions		Preneutral, mean (SE)	Precongruent, mean (SE)	Preincongruent, mean (SE)	Postneutral, mean (SE)	Postcongruent, mean (SE)	Postincongruent, mean (SE)
**RT**							
	Rest	643.6 (44.6)	679.4 (42.6)	732.7 (48.4)	602.5 (27.0)	676.4 (49.4)	693.8 (45.5)
	2D-EX^c^	605.4 (37.4)	637.3 (46.1)	684.3 (58.3)	607.5 (45.1)	637.6 (49.3)	698.6 (67.8)
	VR-EX^d^	602.1 (31.2)	663.1 (35.3)	666.5 (45.0)	597.7 (49.4)	615.0 (40.2)	658.8 (50.4)
**Correct rate**							
	Rest	96.8 (1.2)	96.4 (1.2)	93.2 (2.0)	96.9 (1.2)	96.9 (1.4)	92.7 (2.2)
	2D-EX	97.9 (1.2)	95.3 (1.7)	93.2 (2.1)	96.4 (1.4)	95.3 (1.7)	97.4 (1.6)
	VR-EX	99.0 (0.7)	96.4 (0.9)	93.2 (2.1)	96.4 (1.2)	97.4 (1.2)	96.9 (1.6)

^a^RT: reaction time.

^b^CWST: color-word Stroop task.

^c^2D-EX: exercise in front of a display condition.

^d^VR-EX: exercise with a head-mounted display condition.

### Association Between Executive Performance and Physiological and Psychological Results

**Figure 5:**
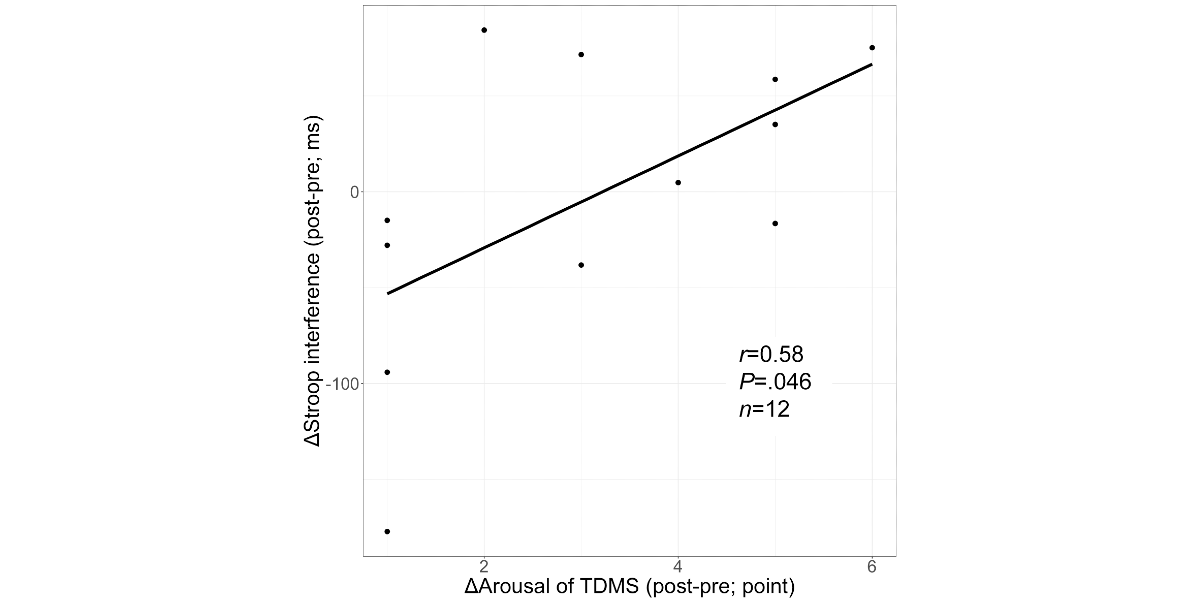
The association between Δarousal (post-pre) and ΔStroop interference (post-pre) in the VR-EX condition. TDMS: Two-Dimensional Mood Scale; VR-EX: exercise with a head-mounted display condition.

We examined the correlation between the change in Stroop interference–related RT and altered physiological and psychological parameters in the 2D-EX and VR-EX conditions. The VR-EX condition showed a significant correlation between Stroop interference and arousal (*r*=0.58, *P=*.046; Figure 5). In the 2D-EX condition, there was no correlation between Stroop interference and any parameters. Furthermore, neither condition showed a correlation between the exercise-induced change in Stroop interference and sEBR before exercise.

### Association Between Physiological and Psychological Results

**Figure 6:**
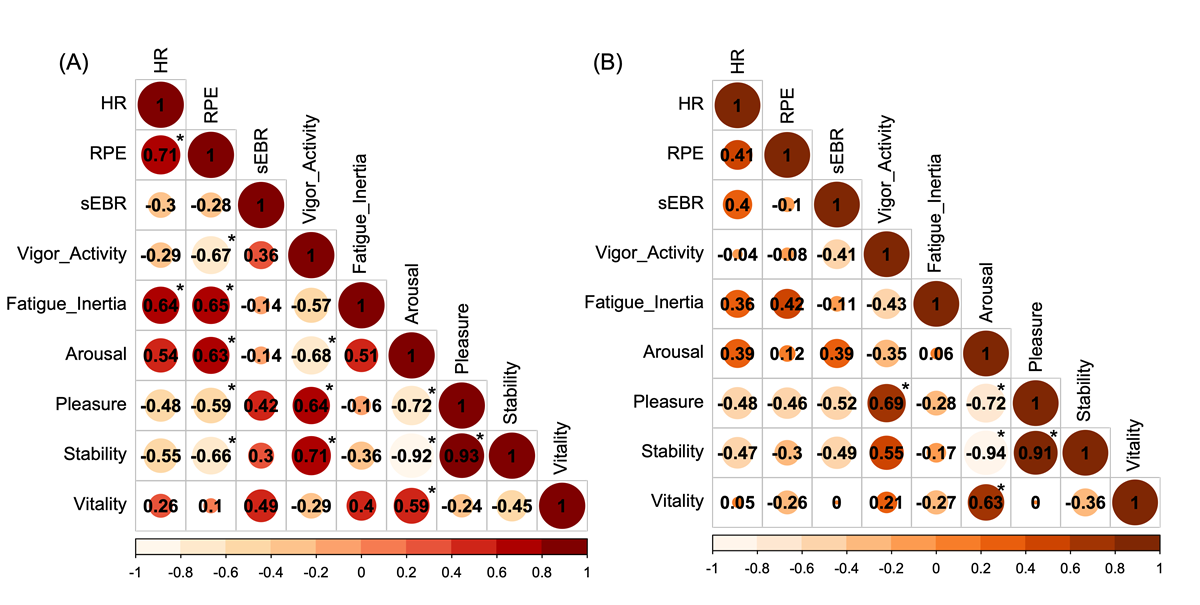
The correlation matrix of physiological and psychological parameters in the (A) VR-EX and (B) 2D-EX conditions. The numbers in the matrix indicate the correlation coefficients between each variable, with the asterisk indicating significant correlations (*P*<.05). Color intensity indicates positive and negative correlation coefficients, and the size of the circle indicates the strength of r. 2D-EX: exercise in front of a display condition; HR: heart rate; RPE: Rating of Perceived Exertion; sEBR: spontaneous eye blink rate; VR-EX: exercise with a head-mounted display condition.

We examined the correlation between physiological and psychological parameters in the 2D-EX and VR-EX conditions. The correlation matrix summarizing the correlation coefficients and significance results between each variable in the VR-EX and 2D-EX conditions is shown in Figure 6. The VR-EX condition showed correlations between HR and fatigue-inertia (*r*=0.64, *P*=.02), RPE and vigor-activity (*r*=*–*0.67, *P*=.02), fatigue-inertia (*r*=0.65, *P*=.02), arousal (*r*=0.63, *P*=.02), pleasure (*r*=*–*0.59, *P*=.04), and stability (*r*=–0.66, *P*=.02). The 2D-EX condition showed no correlations between physiological and psychological parameters. sEBR was not related to any of the psychological parameters in either condition.

## Discussion

### Principal Findings

This study investigated whether VR exergaming enhances executive function and mood. The findings showed that VR exergaming enhanced positive moods, such as vigor-activity and vitality, but did not improve executive function. These results might be explained by the increased arousal level after exercise.

Using estimates of traditional predicted maximum HR [44], we calculated the percent maximum HRs during exercise as 54.1% (2D-EX) and 58.8% (VR-EX). In addition, RPE, which measures a psychological evaluation of exercise intensity, increased to the same extent as in previous studies using a 10-minute moderate-intensity exercise intervention [26,34]. These results suggest that the 2D-EX and VR-EX conditions in this study could be considered as moderate-intensity exercises [45]. As there was no difference in HR between conditions, we believe that the conditions induced the same exercise intensity.

The VR-EX condition caused an increase in the POMS2 vigor-activity scale. Both the 2D-EX and VR-EX conditions increased TDMS arousal, stability, and vitality levels, but arousal and vitality levels differed between the 2 conditions. Participants performed the same-intensity exercise in both conditions, suggesting that the VR exposure might have synergistically increased arousal and vitality levels. These results replicate previous findings that VR exercise improves mood [16]. Conversely, no change in pleasure level was observed in either the 2D-EX or VR-EX condition. As the VR-EX condition involves exergaming to music, we hypothesized that the pleasure level would increase after exercise, because previous studies found that the combination of music and exercise increased the pleasure level [26]; however, this hypothesis was not supported. Although no study participants exhibited symptoms or self-reported VR sickness, it is possible that discomfort from the HMD affected their pleasure level [46,47]. These results might demonstrate the unique characteristics of VR as an exercise environment.

We checked whether Stroop interference, which assesses executive function, was occurring in this experiment. The behavioral measurements revealed a shorter RT in the neutral trials than in the incongruent trials. Thus, we confirmed that Stroop interference could be induced before and after an acute bout of exercise or rest in all conditions. Based on these results, we first compared the effect of all conditions on Stroop interference and found no significant change between conditions; besides, there was no improvement in executive function in either the 2D-EX or VR-EX condition. In addition, there were no differences in executive function improvement between the VR-EX and 2D-EX conditions, despite differences in vigor, arousal, and vitality levels. These results were contrary to our hypothesis and differed from previous studies showing that 10 minutes of moderate-intensity exercise enhanced executive function [30]. Therefore, we examined the physiological and psychological indicators that influenced the changes in executive function in this study.

Our analysis showed an increase in arousal levels as measured by the TDMS in both the 2D-EX and VR-EX conditions, but contrary to previous studies, no association was found in the 2D-EX condition and the opposite association was found in the VR-EX condition. This phenomenon may be considered from the inverse U-shaped model of arousal levels [48]. VR has been shown to increase emotional arousal recorded by multichannel electroencephalograms [49,50], which is consistent with our results showing that the VR-EX condition had a greater arousal increase than the 2D-EX condition. Although exercise-induced increases in intraparticipant arousal levels measured by the TDMS have been associated with enhanced cognitive function and prefrontal activity, which controls cognitive processing [25], excessively increased arousal levels related to high intensity exercise [51] or stress [52] may counteract the beneficial effects of exercise on cognitive function. Therefore, the combination of exercise and VR in this study might have excessively increased arousal levels, counteracting the beneficial effect of exercise on executive function.

Improved executive function from exercise is not only related to arousal level but also to pleasure level [26]. The brain dopaminergic system is related to executive function through prefrontal cortex functions [21-23]. We hypothesized that VR increased the pleasure level and executive function by activating the brain dopaminergic system during exercise. However, our findings showed no change in pleasure levels measured by the TDMS and sEBR (a noninvasive brain dopaminergic system indicator) in the VR-EX condition. This may be because of participants’ lack of familiarity with the exercise style and VR exergaming. No study participants had prior experience with VR exergaming. In addition, many previous studies in which exercise under VR elicited positive mood used bicycle exercises [16-18]; however, participants are more likely to be familiar with a bicycling exercise style than the boxing motion exercise used in this study. Future studies should thus examine long-term intervention effects to familiarize participants with VR and exercise style prior to the VR exergaming intervention.

Furthermore, our findings showed no improvement in executive function in either the VR-EX or 2D-EX condition. The 2D-EX condition in this study combined exercise and computer games, where participants exercised according to the target shown on the display. Therefore, our results are consistent with those of previous studies reporting that a combination of transient exercise and games did not improve executive function [53,54]. Dual tasking has been shown to cause psychological fatigue and cognitive decline (cognitive fatigue) as physical and mental cognitive demands increase [54-57]. However, previous studies in which transient exercise improved executive function have involved only simple bicycle or running exercises [25,26,28,30]. Therefore, the 2D-EX and VR-EX conditions might have increased cognitive demands during exercise, causing cognitive fatigue, which may have counteracted the effect of the exercise on improving executive function. Further research is needed to more comprehensively investigate exercise combined with VR; it is also necessary to examine an exercise-alone condition.

### Limitations

Several limitations of this study should be noted. First, it included only healthy adults without regular exercise habits; thus, there was insufficient interparticipant evaluation. Further validation with athletes, children, and the elderly is needed to clarify the effectiveness of VR exergaming. Second, the TDMS, which is a psychological rather than a physiological scale, was used to assess brain arousal levels. Future studies should assess physiological arousal using electroencephalograms to clarify optimal VR exergaming conditions that enhance brain function based on brain arousal level. Third, this study used FitXR, a VR exergame with a set exercise intensity; it is unclear to what extent the study results generalize to other games. Our findings suggest that games with high cognitive demands might eliminate the positive effect of exercise on executive function; however, further research is needed to determine whether games with low cognitive demands improve executive function. In addition, as mentioned earlier, we examined the transient intervention effects of VR exergaming; however, long-term intervention effects remain unclear. VR exergaming can cause negative effects, such as VR sickness [46], and positive effects, such as improved mood. It is thus necessary to identify the effects of long-term VR exergaming interventions on the mind and body to develop a strategy for promoting physical activity.

### Conclusions

Our study confirms that VR exergaming improves some mood items, but did not provide evidence that it improves executive function. We found that VR exergaming with high cognitive demands may inhibit the positive effects of exercise on executive function. To propose VR exergaming that promotes exercise habituation, it is essential to further examine the combination of exercise and VR conditions that enhances both brain function and mood.

## Abbreviations

2D-EX: exercise in front of a display condition
CWST: color-word Stroop task
HMD: head-mounted display
HR: heart rate
POMS2: Profile of Mood States second edition
RPE: Rating of Perceived Exertion
RT: reaction time
sEBR: spontaneous eye blink rate
TDMS: Two-Dimensional Mood Scale
VR: virtual reality
VR-EX: exercise with a head-mounted display condition
